# The Dispersal Ecology of Rhodesian Sleeping Sickness Following Its Introduction to a New Area

**DOI:** 10.1371/journal.pntd.0002485

**Published:** 2013-10-10

**Authors:** Nicola A. Wardrop, Eric M. Fèvre, Peter M. Atkinson, Susan C. Welburn

**Affiliations:** 1 Geography and Environment, University of Southampton, Highfield Campus, Southampton, United Kingdom; 2 Institute of Infection and Global Health, University of Liverpool, Leahurst Campus, Neston, United Kingdom; 3 School of Biomedical Sciences, University of Edinburgh, Chancellors Building, Edinburgh, United Kingdom; IRD/CIRDES, Burkina Faso

## Abstract

Tsetse-transmitted human and animal trypanosomiasis are constraints to both human and animal health in sub-Saharan Africa, and although these diseases have been known for over a century, there is little recent evidence demonstrating how the parasites circulate in natural hosts and ecosystems. The spread of Rhodesian sleeping sickness (caused by *Trypanosoma brucei rhodesiense*) within Uganda over the past 15 years has been linked to the movement of infected, untreated livestock (the predominant reservoir) from endemic areas. However, despite an understanding of the environmental dependencies of sleeping sickness, little research has focused on the environmental factors controlling transmission establishment or the spatially heterogeneous dispersal of disease following a new introduction. In the current study, an annually stratified case-control study of Rhodesian sleeping sickness cases from Serere District, Uganda was used to allow the temporal assessment of correlations between the spatial distribution of sleeping sickness and landscape factors. Significant relationships were detected between Rhodesian sleeping sickness and selected factors, including elevation and the proportion of land which was “seasonally flooding grassland” or “woodlands and dense savannah.” Temporal trends in these relationships were detected, illustrating the dispersal of Rhodesian sleeping sickness into more ‘suitable’ areas over time, with diminishing dependence on the point of introduction in concurrence with an increasing dependence on environmental and landscape factors. These results provide a novel insight into the ecology of Rhodesian sleeping sickness dispersal and may contribute towards the implementation of evidence-based control measures to prevent its further spread.

## Author Summary

Rhodesian sleeping sickness is a deadly disease, which is spread by the bite of infected tsetse flies in sub Saharan Africa. Rhodesian sleeping sickness has been spreading into new areas of Uganda over the past 15 years; this has been linked to the movement of infected, untreated livestock (which can carry the disease). Until now, there has been little focus on the impact of environmental factors (e.g. land cover) on the spatial spread of sleeping sickness after its introduction to a new area. This work makes use of Rhodesian sleeping sickness case information from December 1998 to November 2002, from an area of Uganda which was not previously affected by the disease, along with the same number of non-sleeping sickness patients, for comparison. Relationships between Rhodesian sleeping sickness and selected factors, including elevation and land cover, were detected using statistical methods. These relationships changed over time, indicating that the disease spread into more ‘suitable’ areas over time, determined by landscape factors. These results provide a novel insight into the environmental factors that guide the spread of Rhodesian sleeping sickness following its introduction to a new area, and can help us to develop more effective control measures to prevent further spread.

## Introduction

Since 1987, the fatal, tsetse-transmitted parasitic disease, Rhodesian sleeping sickness, has spread to eight previously unaffected districts within Uganda [1]–[4]. Recent research has implicated the unrestricted long-distance movement of infected, untreated livestock from endemic areas as being responsible for this spread [5], [6]. It has also been suggested that tsetse movements may have contributed to the spatial spread of Rhodesian sleeping sickness in Uganda [7], [8]. Several steps are required for successful parasite invasion following this type of introduction: establishment of the local population, an increase in parasite abundance and subsequent spatial dispersal [9]–. Disease introduction, each subsequent invasion stage and, therefore, the probability of invasion success, can be influenced by spatial heterogeneity in habitat suitability, environmental conditions and species diversity.

Due to the links between disease transmission and environmental factors, any disruption to a natural ecosystem can have detrimental effects in terms of disease spread, (re)emergence or transmission intensity [12]. Historically, large sleeping sickness epidemics have resulted from ecosystem disruption (large scale cattle depopulation due to Rinderpest [13], and periods of political and civil instability [14]). In contrast, the opposite may be true in some areas: for example, Gambian sleeping sickness has disappeared from several historical foci of disease in west Africa [15]. Dramatic ecosystem changes have occurred within Uganda over the past 50 years, with the virtual elimination of wildlife from large tracts of the country and concurrent increases in human and livestock population densities (wildlife and livestock are reservoirs for *T. b. rhodesiense*). As a result, livestock are now the major reservoir of *T. b. rhodesiense* in Uganda as few other competent host species (or tsetse blood meals) remain. This concentration of circulating parasites in a narrow host range, with which human populations come into regular contact, has likely contributed to an increased risk of transmission to humans and increased vulnerability of unaffected areas to introduction of the disease.

Following parasite introduction, as well as depending on a connectable network of suitable hosts, the probability of successful invasion depends on the suitability of the surrounding landscape [16]. The probability of a successful invasion is increased by spatial dispersion away from the site of introduction, particularly when guided by environmental cues into more suitable habitats that enhance the fitness of the organism in terms of survival and reproduction [9], [11]. The influence of environmental and climatic factors, including proximity to specific types of land cover (specifically areas of wetland), on the spatial distribution of tsetse vectors and Rhodesian sleeping sickness has been demonstrated [5], [17]–[19]. Although previous research has indicated a temporal trend in the associations between Rhodesian sleeping sickness and landscape factors following the introduction of the disease to a new area [20], the significance of such factors on the probability of *T. b. rhodesiense* introduction success and its subsequent spatial dispersal has not been investigated, despite the potential practical applications of such knowledge for targeted disease control activities. A wealth of information is available regarding the impact of environmental heterogeneity on the invasion and spread of exotic plants and animals [21], [22]. Despite clear overlaps with the ecology of animal and plant invasions, much of the literature relating to the spatially heterogeneous dispersal of infectious diseases is focused on population dynamics and social interactions, largely ignoring the contribution of environmental variables [10], [23], [24]. An understanding of the spatio-temporal interplay between parasite transmission and the natural environment would provide important information for the prevention of future spread linked to new introductions.

We used annually stratified data to examine the spatio-temporal evolution of a Rhodesian sleeping sickness outbreak within an area of eastern Uganda which was unaffected by the disease until 1998. A land cover classification was used to characterise components of the landscape which may influence the distribution of tsetse and, thus, the spread of sleeping sickness. Temporal changes in the observed relationships between sleeping sickness and selected landscape factors were studied to provide an understanding of the factors influencing the dispersal of sleeping sickness following its introduction. This research acts as an initial bridge between the invasion ecology of animals, plants and infectious diseases, emphasising the importance of multi-faceted ecosystem attributes on the probability of transmission establishment and dispersal. The analysis can provide important information to assist with the prevention of future transmission establishment and dispersal of disease following an introduction.

## Materials and Methods

### Ethics statement

No patient identifiable information was recorded to maintain patient confidentiality and to adhere to the International Ethical Guidelines for Biomedical Research Involving Human Subjects. The use of these data was approved by the University of Edinburgh Research Ethics Committee. Informed consent was not obtained as secondary data were used, and all data were anonymous.

### Study area

**Figure 1:**
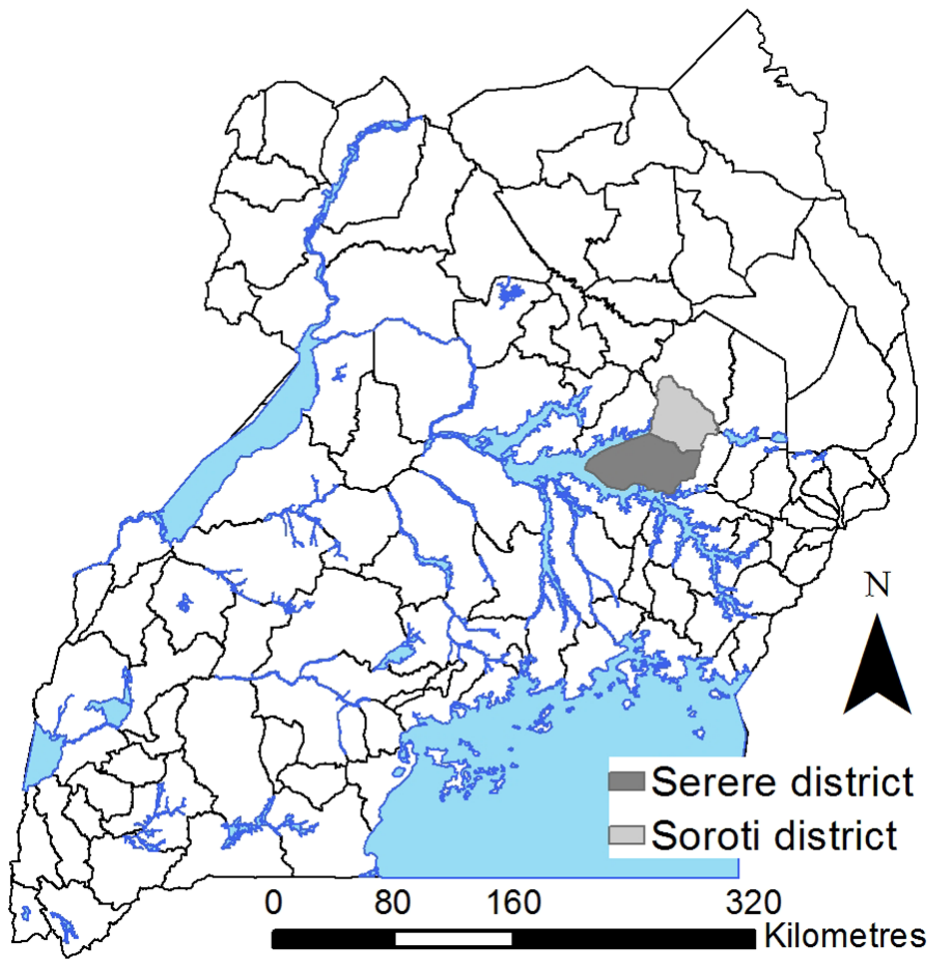
Map of Uganda, highlighting the study area (Serere and Soroti districts). Note that Serere district was part of Soroti district prior to 2010.

The research focused on the catchment of Serere Hospital (formerly Serere Health Centre) in Serere district (formerly part of Soroti District), which is in the Eastern region of Uganda and borders Lake Kyoga (see Figure 1 for a map of Uganda highlighting the study area). The population within the study district during the 2002 national census, predominantly composed of the Iteso and Kumam ethnic groups, was approximately 104,400, with a population density of approximately 89 people per km^2^ [25]. Primary economic activities within the study area are subsistence farming (predominantly rain-fed) and fishing (in areas in close proximity to the lake) [26]. The cattle density within the study area was approximately 105 per km^2^ in the 2008 livestock census, although this is likely to be higher than the cattle density during the study period (1998 to 2002) due to on-going cattle restocking activities [27]. The main livestock market in the area is Brookes Corner livestock market, which runs on a weekly basis, trading an average of 90 to 120 cattle per week.

### Case control data

A matched case-control study design was used with 1∶1 matching. Passively detected Rhodesian sleeping sickness case records from Serere Hospital were used as cases. Each case was matched to a suitable control from the hospital inpatient, outpatient, tuberculosis and maternity ward records (in that order of preference), excluding patients with a primary diagnosis of a vector-borne disease (to prevent spatial bias in the results). Matching was carried out based on age group (<1, 1–9, 10–14, 15–19, 20–49, 50–64 and ≥65 years), gender and month of admission. The matched study design was used to ensure that the controls were selected from the same population as the cases, thus, avoiding spatial bias. The case-control data covered the time period from December 1998 to November 2002. A hand-held global positioning system (GPS) (Garmin, Olathe, KS) was used to record the location at each village's central meeting point, as determined by the village chairperson. Part of the dataset (up to June 2000) was used to identify the local livestock market as the original point of introduction [2].

### Covariate data

A land cover classification was carried out using Landsat Enhanced Thematic Mapper plus (ETM+) images from 2001 in the eCognition software Version 4.2 (Definiens, Munich, Germany) [28]. The classification defined areas of “seasonally flooding grassland” and “woodland and dense savannah”, which were of interest due to the habitat requirements (prefers riverine vegetation) of the predominant vector species in the study area; *Glossina fuscipes fuscipes*. More details for the land cover classification are given in the supplementary information.

The geo-referenced case-control data were visualised using ArcMap 9.1 (ESRI, Redlands, CA) along with the land cover classification. Circular buffers of 1 km (to represent the majority of the village area) and 3 km radii (to include the areas immediately surrounding the village) were created around each village centroid. This choice of buffer size was based on previous research demonstrating the significance of wetland areas up to 3 km from the homestead for the risk of Rhodesian HAT [29]. The proportions of pixels within the buffer areas that were classified as “seasonally flooding grassland” or “woodland and dense savannah” were calculated to quantify the landscape within and surrounding the case and control villages. Population density [30], elevation [31] and predicted suitability (as a percentage) for *G. f. fuscipes*, the predominant vector species within the study area [32], were overlaid with the case-control data in ArcMap and their values extracted for each case-control observation. The Euclidean distance (kilometres) between each village and Brookes Corner livestock market (previously implicated in the introduction of Rhodesian sleeping sickness to the study area [2]), was calculated.

### Exploratory analysis

The dataset was stratified annually to allow the separate analysis of each year and illustrate temporal patterns in the observed relationships. The four years for analysis ran from December to November and, for clarity, will be referred to as the first (December 1998 to November 1999), second (December 1999 to November 2000), third (December 2000 to November 2001) and fourth (December 2001 to November 2002) years. Unadjusted odds ratios were calculated to examine the relationships between sleeping sickness status (case or control) and each independent variable for the four years using conditional logistic regression (a variation of logistic regression which takes into account a matched study design [33], [34]) implemented using the *Survival* package in R statistical software [35]. The odds ratios were plotted over time to highlight temporal changes.

### Multivariate analysis

Multivariate conditional logistic regression analysis was carried out for each of the four years, using forwards stepwise methodologies, beginning with the null model in each year. At each step, the variable which resulted in the largest decrease in deviance was selected. Models were compared using Chi-squared likelihood ratio tests, and variables were retained in the model if this test was significant, and the variable was significant within the model (*p*-value<0.05). Any variables which lost significance in subsequent steps were removed. The stepwise addition of interaction terms between the significant variables was carried out to assess the presence of effect modification. *R* ^2^ values were used to assess the proportion of the variability in the response variable which was described by the model (note: the maximum *R* ^2^ for conditional logistic regression is 0.5). Predicted suitability for *G. fuscipes* was excluded from the multivariate analysis as this variable is itself a predictive model output encompassing the effects of several covariates.

## Results

**Figure 2:**
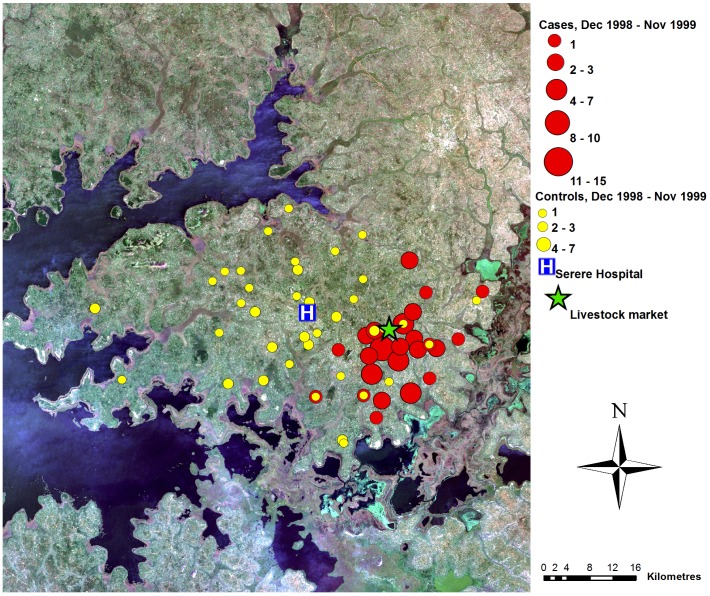
Map of case control data, year 1. True colour Landsat ETM+ composite of study area, showing locations of cases and controls (as counts for each location), December 1998–November 1999, Serere hospital and Brookes Corner livestock market. Figure adapted from [20].

**Figure 3:**
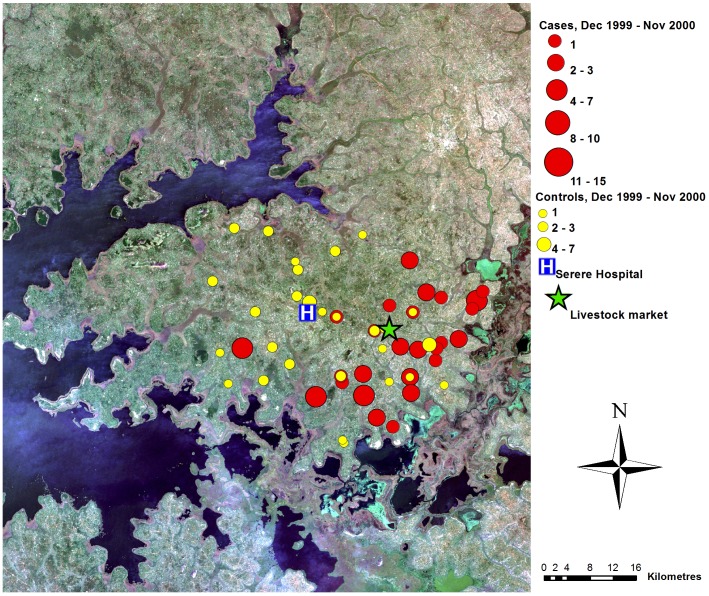
Map of case control data, year 2. True colour Landsat ETM+ composite of study area, showing locations of cases and controls (as counts for each location), December 1999–November 2000, Serere hospital and Brookes Corner livestock market. Figure adapted from [20].

**Figure 4:**
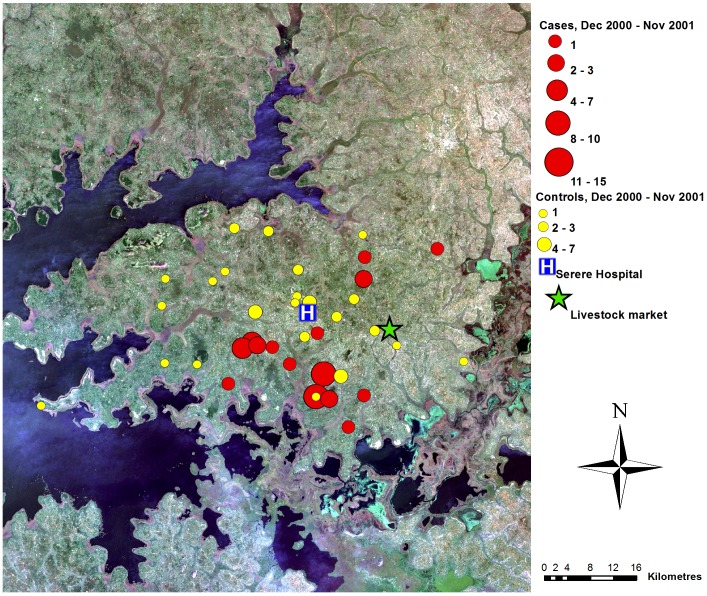
Map of case control data, year 3. True colour Landsat ETM+ composite of study area, showing locations of cases and controls (as counts for each location), December 2000–November 2001, Serere hospital and Brookes Corner livestock market. Figure adapted from [20].

**Figure 5:**
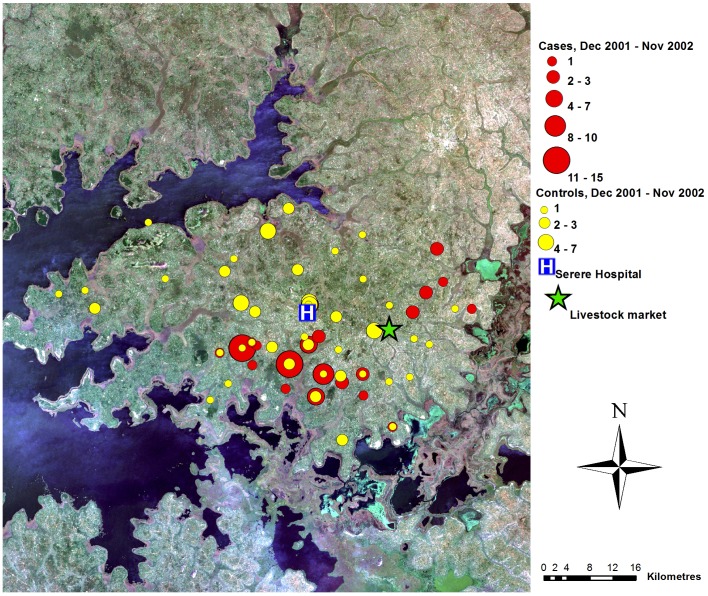
Map of case control data, year 4. True colour Landsat ETM+ composite of study area, showing locations of cases and controls (as counts for each location), December 2001–November 2002, Serere hospital and Brookes Corner livestock market. Figure adapted from [20].

A total of 240 sleeping sickness cases resident within the study area were diagnosed at Serere hospital during the study period (December 1998 to November 2002). Suitable controls could not be identified for seven cases; these were excluded (see Table 1 for the number of matched and unmatched cases from each of the four years). The spatial distribution of the 233 cases within the study area varied through the study period (Figures 2 to 5). During the first year, the cases were primarily located close to the livestock market where the parasite has previously been demonstrated to have been introduced via the trade of untreated cattle, and not far from one of the main tributaries of like Kyoga. In contrast, the controls were evenly distributed across the study area. Over the subsequent years, the distribution of controls remained evenly dispersed with no directional movement, while the spatial distribution of cases moved away from the livestock market.

**Table 1:** Number of matched and unmatched cases for each year.

Time period	Number of unmatched cases	Number of matched cases
December 1998 to November 1999	2	58
December 1999 to November 2000	4	52
December 2000 to November 2001	1	44
December 2001 to November 2002	0	79
Total	7	233

### Univariate analysis

**Figure 6:**
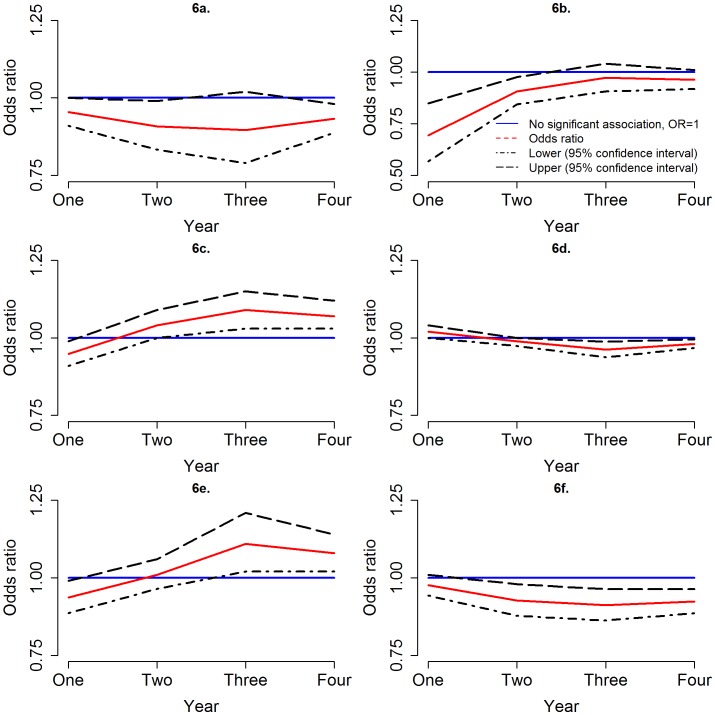
Temporal trend in odds ratios. Odds ratios (red line) over the four years for (a) population density (top left), (b) distance to livestock market (top left), (c) predicted suitability for *G. fuscipes* (centre left), (d) elevation (centre right), (e) proportion of land within 3 km that is seasonally flooding (bottom left) and (f) proportion of land within 1 km that is “woodland and dense savannah” (bottom right), with 95% confidence interval for the odds ratio (broken line) in comparison with an odds ratio of 1, which represents no significant association (blue line). Note that 2 (b) has a different scale on the y-axis from the other plots.

All four non-land cover variables (elevation, population density, predicted suitability for *Glossina fuscipes fuscipes* and distance to the local livestock market) were significantly correlated with the occurrence of sleeping sickness; temporal trends in the odds ratios were observed for three of them (see Table 2 and Figures 6a to 6d). Increasing population density demonstrated a protective effect in each of the four years (Odds ratios (OR) = 0.95, 0.91, 0.90 and 0.93 for years one to four respectively - this was not statistically significant in the third year). A protective effect was also observed at increasing distances from the livestock market during all four years (OR = 0.69, 0.91, 0.97 and 0.96 for years one to four respectively), although this correlation lost statistical significance in the third and fourth years as the strength of the association decreased. In the first year, areas with a higher predicted suitability for *G. f. fuscipes* had a significantly decreased odds of sleeping sickness occurrence (OR = 0.95), but in the years two, three and four the correlation reversed (OR = 1.04, 1.09 and 1.07 respectively). A similar temporal pattern was observed for elevation, with an increased odds of sleeping sickness in higher elevation areas in the first year (OR = 1.02), and decreased odds in the second to fourth years (OR = 0.99, 0.96 and 0.98, respectively, although this was not statistically significant in the second year).

**Table 2:** Univariate results for the four years.

Variable		Year one	Year two	Year three	Year four
Population density	**OR ** ***(95% CI)***	0.95 *(0.91–1.00)*	0.91 *(0.83–0.99)*	0.90 *(0.79–1.02)*	0.93 *(0.89–0.98)*
	***p*** **-value**	0.05	0.03	0.09	0.006
Predicted suitability for *G. fuscipes*	**OR ** ***(95% CI)***	0.95 *(0.91–0.99)*	1.04 *(1.00–1.09)*	1.09 *(1.03–1.15)*	1.07 *(1.03–1.12)*
	***p*** **-value**	0.01	0.05	<0.005	<0.005
Elevation	**OR ** ***(95% CI)***	1.02 *(1.00–1.04)*	0.99 *(0.97–1.00)*	0.96*(0.94–0.99)*	0.98 *(0.97–0.99)*
	***p*** **-value**	0.03	0.15	<0.005	0.008
Distance to livestock market	**OR ** ***(95% CI)***	0.69 *(0.57–0.85)*	0.91 *(0.84–0.98)*	0.97 *(0.91–1.04)*	0.96 *(0.92–1.01)*
	***p*** **-value**	<0.005	0.009	0.44	0.11
“woodland and dense savannah” (1 km)+	**OR (95% CI)**	0.98 *(0.94–1.01)*	0.93 *(0.88–0.98)*	0.91 *(0.86–0.96)*	0.92 *(0.89–0.96)*
	**p-value**	0.16	0.008	<0.005	<0.005
“Seasonally flooding grass” (3 km)++	**OR (95% CI)**	0.94 *(0.89–0.99)*	1.01 *(0.97–1.06)*	1.11 *(1.02–1.21)*	1.08 *(1.02–1.14)*
	**p-value**	0.02	0.69	0.02	0.007

Unadjusted odds ratios (OR), 95% confidence intervals (CI) and *p-*vales where calculated using conditional logistic regression.

+ Proportion of land within 1 km which is “woodland and dense savannah.”

++ Proportion of land within 3 km which is “seasonally flooding grassland.”

We considered the land cover in the areas surrounding case and control villages. Significant correlations were found between sleeping sickness occurrence and the proportions of “seasonally flooding grassland” and “woodland and dense savannah” within 1 km and 3 km buffers around villages. In year one, villages with a higher proportion of “seasonally flooding grassland” in the surrounding areas had lower odds of sleeping sickness occurrence, but in years two to four the relationship reversed. This correlation had increased statistical significance using a buffer of 3 km radius (OR = 0.94, 1.01, 1.11 and 1.08 for years one to four, respectively, although this was not statistically significant in year two). The presence of a higher proportion of “woodland and dense savannah” within areas surrounding the villages demonstrated a protective effect, with increased significance for the 1 km radius buffers (OR = 0.98, 0.93, 0.91 and 0.92 for years one to four respectively, although this was not statistically significant for year one).

### Multivariate analysis

Multivariate conditional logistic regression resulted in three different final regression models (see Table 3). The model for years one and two contained distance to the livestock market (OR = 0.48, *p*<0.005 for year one, OR = 0.79, *p*<0.005 for year two), which had a protective effect at greater distances (the effect was smaller in the second than the first year), and elevation (OR = 0.91, *p* = 0.02 for year one, OR = 0.95, *p*<0.005 for year two) which demonstrated a protective effect in areas with higher elevation. The final model for year three included the proportion of “woodland and dense savannah” within a 1 km buffer (OR = 0.88, p<0.005) which demonstrated a protective effect in areas with a higher proportion, and the proportion of “seasonally flooding grassland” within a 3 km buffer (OR = 1.18, p = 0.01) which resulted in an increased odds of sleeping sickness in areas with a higher proportion. For the fourth year, the final model contained the proportion of “woodland and dense savannah” within a 1 km buffer (OR = 0.90, p<0.005) which demonstrated a protective effect in areas with a higher proportion, and distance to the livestock market (OR = 0.90, p = 0.01), which resulted in lower odds of sleeping sickness occurrence in areas further from the market (this effect was smaller in year four than years one or two). The *R* ^2^ for each model differed, with the largest *R* ^2^ for year one (0.41 compared with 0.21, 0.26 and 0.18 for years two to four, respectively).

**Table 3:** Final multivariate models for each of the four years.

Year	Variable	OR *(95% CI)*	*p* - value
One	Distance to market	0.48 *(0.30–0.77)*	<0.005
	Elevation	0.91 *(0.84–0.99)*	0.02
	* *R* ^2^ = 0.41, AUC = 0.99		
Two	Distance to market	0.79 *(0.68–0.90)*	<0.005
	Elevation	0.95 *(0.93–0.98)*	<0.005
	* *R* ^2^ = 0.21, AUC = 0.86		
Three	Proportion woodland (1 km)	0.88 *(0.81–0.95)*	<0.005
	Proportion flooding (3 km)	1.18 *(1.04–1.33)*	0.01
	* *R* ^2^ = 0.26, AUC = 0.90		
Four	Proportion woodland (1 km)	0.90 *(0.85–0.95)*	<0.005
	Distance to market	0.90 *(0.83–0.98)*	0.01
	* *R* ^2^ = 0.18, AUC = 0.84		

Odds ratios (OR), 95% confidence intervals (CI) and *p-*values, *R* ^2^ values and the area under the receiver operating characteristic curve (AUC).

* Note that the maximum *R* ^2^ value in a conditional logistic regression model is 0.5.

## Discussion

The temporal analysis identified the influence of selected landscape factors on the spatially heterogeneous dispersal of Rhodesian sleeping sickness following its introduction to a previously unaffected area. We analysed four annual snapshots to show the nature of the spatial dispersal of Rhodesian sleeping sickness into the landscape over time, following introduction via a cattle market. This dispersal of disease outcomes is seen as a manifestation of an underlying ecological dispersal process of the Trypanosome parasite into suitable areas conditional upon landscape suitability, cattle density and movements, human density and movements and the spatial distribution of tsetse populations. We quantified the indirect dependence of disease outcomes on non-landscape constraints (markets) and landscape constraints (land cover); and crucially, the switch from the former to the latter spatially and the rate at which this occurs. Proximity to the point of introduction initially exerted a strong spatial control, but this correlation diminished approximately two years after first measurement, in concurrence with an increasing dependence on environmental factors as the disease flowed outward to occupy the most suitable environmental niche.

In line with previous studies, increasing population density demonstrated a protective effect [17], [36], illustrating the preference of the tsetse vector for areas with lower population density and, thus, less disturbance of vegetation and potential habitats or *vice versa* the avoidance of tsetse suitable habitats by humans. However, it must be noted that changes in the population size are likely to have occurred between the study period and the year for which population density data was available (2006). These population changes are not likely to have affected the results, but should be kept in mind. Higher predicted suitability for the vector, *G. fuscipes*, was shown to be a risk factor in years two to four, with a temporal trend in the relationship suggesting that parasite dispersal from the point of introduction may have been into areas more suitable for vector populations and, thus, (where humans and livestock are present) for transmission of sleeping sickness. Distance to the livestock market was negatively correlated with sleeping sickness as has been noted previously [2]. A temporal trend was also evident for this correlation; the strength of the association decreased and lost statistical significance over time, illustrating the spatial dispersal away from the initial point of introduction [2]. The re-inclusion of distance to the livestock market in the multivariate model for year four provides conflicting evidence from the univariate analysis which illustrated the apparent dispersion of cases away from the livestock market over the study period. This may be indicative of a secondary parasite introduction at the livestock market following the initial introduction in (or before) 1998, although it is not possible to confirm this using the data available.

Temporal trends are also apparent in the relationships between sleeping sickness status and selected landscape features. The odds ratios for elevation and the proportion of land surrounding villages that was “woodland and dense savannah” decreased over time (indicating dispersal into lower lying areas with less “woodland and dense savannah”) and the odds ratio for the proportion of “seasonally flooding grassland” surrounding villages increased (indicating dispersal into areas with more “seasonally flooding grassland”). The observed trends suggest that these landscape features contributed to the spatially heterogeneous dispersal away from the point of introduction; the areas into which the disease spread may represent more ‘suitable’ habitats for tsetse survival and reproduction and, thus, sleeping sickness transmission. The observed temporal trend in the correlation with elevation may be related to “seasonally flooding grassland”, as lower lying areas are more likely to become flooded during the wet season. Further factors which may influence the spatial distribution and dispersion of Rhodesian sleeping sickness, but which have not been considered here, include proximity to water bodies, livestock density, climatic factors (although climatic factors are not likely to vary significantly at the scale of the study area) and tsetse abundance (tsetse suitability was considered in univariate, but not multivariate analysis). The available predicted tsetse suitability was not included in multivariate modelling due to the fact that this in itself is the result of a statistical model; no other tsetse abundance data were available for the study area. Movements of humans, livestock and tsetse within the study area are also likely to play an important role in the spatial dispersal of parasites following introduction to a new area. However, data regarding these movements were not available, and, thus, it was not possible to account for this in the analysis. Further development of the analytical framework presented should attempt to incorporate these local movements to provide a broader picture of disease dispersal. Treatment of humans and cattle for trypanosomiasis may have altered the epidemiology of Rhodesian sleeping sickness during the study period, although this is not likely to have influenced the spatial spread, or the correlations between disease occurrence and landscape factors.

This research quantifies the influence of environmental factors on the space-time dynamics of Rhodesian sleeping sickness for the first time and acts as an initial bridge between the invasion ecology of animals and plants (which has been widely studied [21], [22]), and the invasion ecology of infectious diseases (which has, thus far, been largely neglected), emphasising the importance of multi-faceted ecosystem attributes on the probability of disease establishment and dispersal. It is now established that Rhodesian sleeping sickness introductions have occurred via livestock trade into markets in Uganda [2], [5]. The continuing movement of untreated animals through the study area and other currently unaffected areas, in conjunction with large livestock populations and low diversity of alternative hosts, increases the likelihood of further parasite introductions.

Within the wider context of sleeping sickness epidemiology across sub-Saharan Africa, Uganda is one of 36 countries affected by sleeping sickness. Despite the progressive spread of Rhodesian sleeping sickness within Uganda, the annual number of reported cases has been falling in Uganda and elsewhere. Some countries which were previously considered to be endemic for the disease have not reported any cases in recent years, indicating possible local elimination [37], [38]. Despite reductions in annual incidence and the institution of animal based interventions to reduce the reservoir of *T. b. rhodesiense* in northern Uganda (The Stamp Out Sleeping Sickness (SOS) programme which began in 2006 to prevent further spread [39]), there is still concern of further spread: since 2004, cases of Rhodesian sleeping sickness have been reported in further districts of Uganda which were previously not affected, north west of Serere district [5]. Further spread of *T. b. rhodesiense* within Uganda is possible, thus, increased understanding of the process of spatial spread of disease is required to enable effective targeting of interventions.

This analysis serves to enhance our current understanding of the spread of Rhodesian sleeping sickness and other environmentally dependent diseases, and demonstrates, for the first time, through the evolution of sleeping sickness cases, the role played by the landscape on the possibility of Trypanosome dispersal. These findings can provide foresight for intervention and planning: an expectation of the rate and extent to which suitable areas may become affected should allow suitable areas that are proximate to an introduction site to be targeted in advance of disease dispersal. For example, tsetse traps may be utilised within suitable landscapes in close proximity to livestock markets. Future research should extend this analysis to other historical outbreaks of Rhodesian sleeping sickness following introduction and to other diseases where introduction is known to occur through a focal site.

## Supporting Information

Checklist S1 STROBE checklist.
(DOC)

Text S1 Detailed methods for the land cover classification.
(DOC)
