# Supplementary material for: The Dispersal Ecology of Rhodesian Sleeping Sickness Following Its Introduction to a New Area
Source: PLoS Negl Trop Dis. 2013 Oct 10;7(10):e2485. doi: 10.1371/journal.pntd.0002485 (PMC3794918; doi:10.1371/journal.pntd.0002485)
Supplement: Text S1 — Detailed methods for the land cover classification. (DOC) [file pntd.0002485.s002.doc]

**Supplementary methods**

**Image processing**

Three Landsat ETM+ images (path 171, row 59; level 1T products), were used from 27^th^ January, 17^th^ April and 27^th^ November 2001. Atmospheric correction (and haze removal for the April image) was carried out using ATCOR-2 (Atmospheric & Topographic Correction for Small FOV Satellite Images; ReSe Applications Schläpfer, Switzerland). Normalised difference vegetation index (NDVI) was calculated for each image using the following formula: NDVI = (near-infrared – red)/(near-infrared + red)^1^.

Supervised object-based classification

Training polygons for the land cover classes shown in Table S1 were collected by Dr Eric M Fèvre in 2001 using a handheld GPS (Garmin, Olathe, KS) and supplemented using Quick Bird imagery available in Google Earth (Google™, Mountain View, CA) for land cover classes which were easily identifiable and not likely to change over time. A random 20% sample was extracted for validation. Based on feature space plots, class descriptions and prior knowledge, several classes were aggregated prior to classification (see Table S1). Segmentation and classification of the images was carried out using the eCognition software Version 4.2 (Definiens, Munich, Germany). Segmentation was carried out using a scale parameter of 5, homogeneity criteria with 90% emphasis on spectral homogeneity and 10% shape, and 50% for both compactness and smoothness^2^. The classification was performed in two steps (an initial classification and a sub-classification), as shown in Figure S1. For the level 1 classification, “open water” was classified using a threshold value for one band, selected by visual interpretation (Table S2). The remaining level 1 classes (“built up and bare ground”, “dry vegetation types” and “wet vegetation types”) were classified using a nearest neighbour algorithm, utilising the image features as detailed in Table S2, (level 1 classification). The level 2 classification sub-classified the “dry vegetation types” class into “woodland and dense savannah” and “crops and open savannah”, and “wet vegetation types” into “seasonally flooding grassland” and “lake fringe swamps” using nearest neighbour algorithms, utilising the image features as detailed in Table S2 (level 2 classification). The classification was validated using the validation sample, leading to an error matrix. Producer’s and user’s accuracies for each class, plus an overall accuracy value were calculated^3^. The class “open water” was excluded from the validation as the threshold selection was not based on training data.

**Classification results**

The overall classification accuracy was 86%, with producer’s and user’s accuracies of over 70% for all classes except “crops and open savannah” (producer’s accuracy = 69%, user’s accuracy = 47%), which was not thought to be a significant tsetse habitat within the study area. The error matrix is shown in Table S3 and the classification in Figure S2. Within the study area, the predominant land cover classes were “crops and open savannah” (31.4% of the study area), followed by “open water” and “woodland and dense savannah” (both 17.9%). “Seasonally flooding grassland” accounted for 14.3%, “lake-fringe swamps” 11% and “built up and bare ground” 7.5%.

**Covariate extraction**

Circular buffers of 1 km and 3 km radii were created around each village. The radii sizes were based on previous studies investigating the significance of wetlands for sleeping sickness distributions^4^ and the daily mobility patterns of Ugandan village residents^5^. The percentage of the area within each buffer classified as “seasonally flooding grassland” and “woodland and dense savannah” was calculated. Several additional datasets were obtained: population density (expressed as hundreds of people per square kilometre)^6^; elevation^7^ and predicted suitability for *G. f. fuscipes* (as a percentage)^8^. These were overlaid with the case-control data in ArcMap and their values extracted for each case or control. The distance between each case or control and Brookes Corner livestock market (the source of Rhodesian sleeping sickness in the study area^9^) was calculated.

| **Initial land cover class** | **Description** | **Final class** |
| --- | --- | --- |
| Maize | Fields of specific crops | Crops, agricultural and open savannah |
| Cassava |
| Banana and coffee |
| Ground nuts |
| Millet |
| Peas |
| Pineapple |
| Rice |
| Sim sim |
| Ploughed fields | Ploughed fields with no growth |
| Open savannah | Grassland with occasional trees/bushes (not seasonally flooding) |
| Dense savannah | Grassland with dense trees/bushes (not seasonally flooding) | Woodland and dense savannah |
| Deciduous woodland | Patches of deciduous woodland |
| Evergreen woodland | Patches of pine woodland |
| Riverine woodland | Patches of deciduous woodland along rivers |
| Rural built up | Rural towns/villages with high density of buildings | Built up and bare ground |
| Urban | Major town with high density of roads, roofs and covered markets |
| Grass lawns | Large school playing fields (dry grass) |
| Bare ground | Bare murrum or mud |
| Open water | Areas of open water (lake or wide river) | Open water |
| Lake fringe swamps | Lake edges with a high density of papyrus, water hyacinth and water lilies | Lake fringe swamps |
| Seasonally flooded grassland | Savannah which floods during the wet season, with occasional trees/bushes | Seasonally flooded grassland |

**Table S1:** Initial land cover classes (which determined the training data collected by Fèvre, E. M. in the field) along with final aggregated classes used in the land cover classification.


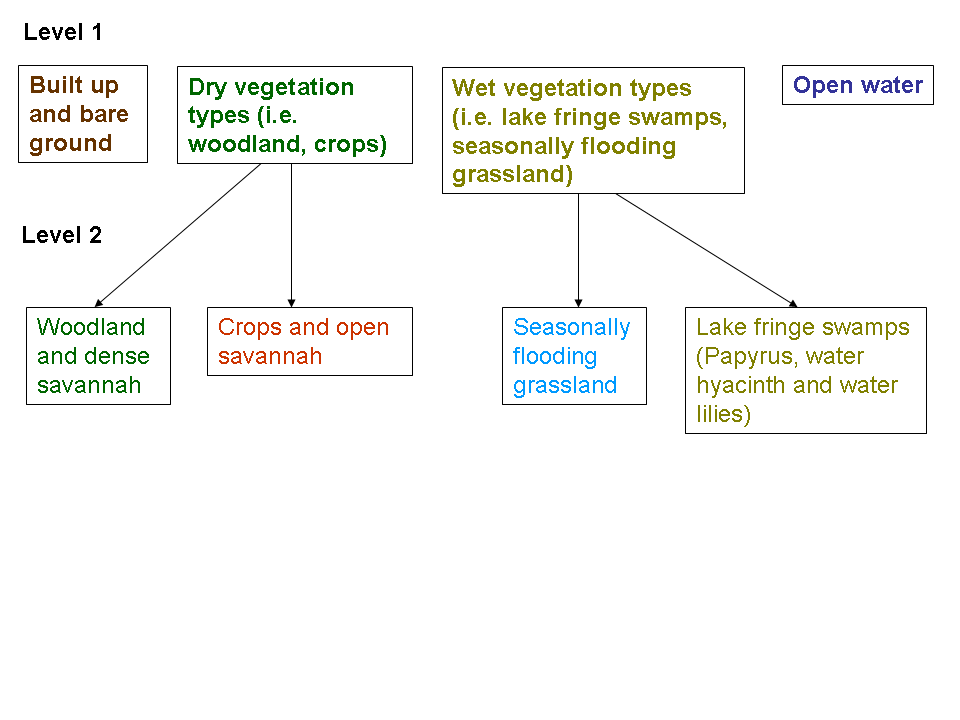


**Figure S1:** Land cover class hierarchy showing super-classes (level 1) and sub-classes (level 2).

|  | **Classes** | **Features** | **Image** | **Notes** |
| --- | --- | --- | --- | --- |
| **Level 1 classification** | Open water | Object mean band 4 (near infrared): threshold of 5.5% | April | Light in band 4 absorbed by water^10^ |
|  | Built up & bare ground; wet vegetation types; dry vegetation types | Object mean band 3 (red) | April | Vegetation type discrimination^11^ |
|  |  | Object mean band 2 (green) | November | Identification of healthy vegetation^11^ |
|  |  | Object mean band 3 | November | Vegetation type discrimination^11^ |
|  |  | Object mean band 5 (mid infrared) | November | Vegetation and soil moisture content^11^ |
|  |  | Object mean band 7 (mid infrared) | November | Identification of built up areas and bare ground^11^ |
|  |  | Object length/width |  |  |
|  |  | NDVI difference, January to November |  | Differentiation of vegetation types |
| **Level 2 classification** | Woodland & dense savannah*; crops & open savannah* | Object mean band 2 | April | Related to healthy vegetation, biomass, plant type or vegetation moisture content^11^ |
|  |  | Object mean band 3 | April |  |
|  |  | Object mean band 4 | April |  |
|  |  | Object mean band 5 | April |  |
|  |  | NDVI difference, January to April, January to November and April to November |  | Differentiation of vegetation types |
|  | Seasonally flooding grassland**; lake fringe swamps** | Object mean band 4 | November | Identification of vegetation and soil moisture content, biomass, plant vigour and water^11^. |
|  |  | Object mean band 5 | November |  |

**Table S2:** Image features used for level 1 and level 2 classifications. All classifications were based on a nearest neighbour algorithm with the exception of open water, which was based on a threshold.

*Obtained using sub-classifications of the “dry vegetation types” class. **Obtained using sub-classifications of the “wet vegetation types” class.


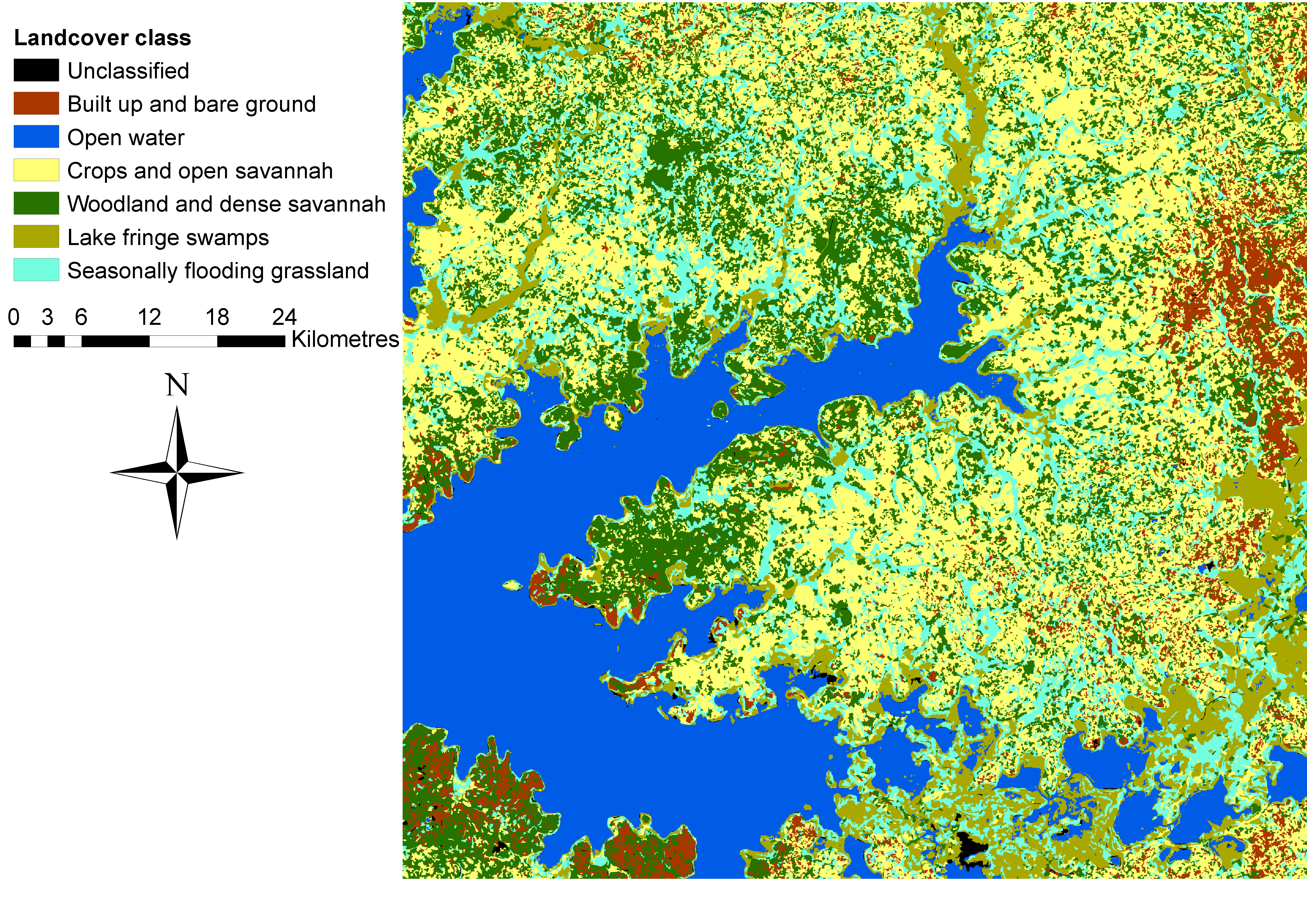


**Figure S2:** Level 2 land cover classification using supervised object-based classification.

|  | | **Actual land cover class** | | | | |  | |
| --- | --- | --- | --- | --- | --- | --- | --- | --- |
|  |  | Built up & bare ground | Crops & open savannah | Woodland & dense savannah | Lake fringe swamps | Seasonally flooding grassland | Total | User’s accuracy |
| **Predicted land cover class** | Built up & bare ground | 56 | 3 | 0 | 0 | 0 | **59** | 94.9% |
|  | Crops & open savannah | 9 | 27 | 22 | 0 | 0 | **58** | 46.6% |
|  | Woodland & dense savannah | 2 | 3 | 222 | 0 | 0 | **227** | 97.8% |
|  | Lake fringe swamps | 0 | 6 | 30 | 111 | 3 | **150** | 74.0% |
|  | Seasonally flooding grassland | 0 | 0 | 14 | 0 | 140 | **154** | 90.9% |
|  | Unclassified | 0 | 0 | 0 | 0 | 0 | 0 |  |
|  | Total | **67** | **39** | **288** | **111** | **143** |  | |
|  | Producer’s accuracy | 83.6% | 69.2% | 77.1% | 100% | 97.9% |  | |

**Table S3:** Error matrix for Level 2 classification. Overall accuracy = 86%

References

^1^ Tucker, C. J., "Red and photographic infrared linear combinations for monitoring vegetation." *Remote Sens. Environ.* **8** , 127 (1979).

^2^ M. Baatz*, et al.*, eCognition professional user guide 4, 2000.

^3^ Lillesand, T. M., Kiefer, R. W., and Chipman, J. W., "Digital Image Processing,"in *Remote Sensing and Image Interpretation,* Fifth ed.(John Wiley and Sons, Hoboken, 2004), pp.491-637.

^4^ Zoller, T., Fèvre, E. M., Welburn, S. C., Odiit, M., and Coleman, P. G., "Analysis of risk factors for T. brucei rhodesiense sleeping sickness within villages in south-east Uganda." *BMC Infect. Dis.* **8** (1), 88 (2008).

^5^ Bryceson, D. F., Mbara, T. C., and Maunder, D., "Livelihoods, daily mobility and poverty in sub-saharan Africa." *Transport Reviews* **23** (2), 177 (2003).

^6^ Oak Ridge National Laboratory, "Landscan 2006 Global Population Dataset,"in (Oak Ridge National Laboratory, 2006).

^7^ US Geological Survey, "Finished" 3-arc second SRTM Format Documentation, 2006.

^8^ W. Wint and D. J. Rogers, Predicted distributions of tsetse in Africa, 2000.

^9^ Fevre, E. M., Coleman, P. G., Odiit, M., Magona, J. W., Welburn, S. C., and Woolhouse, M. E. J., "The origins of a new Trypanosoma brucei rhodesiense sleeping sickness outbreak in eastern Uganda." *Lancet* **358** (9282), 625 (2001).

^10^ Campbell, J. B., "Hydrospheric Sciences,"in *Introduction to Remote Sensing,* Third ed.(The Guildford Press, New York, 2002), pp.523-553.

^11^ Lillesand, T. M., Kiefer, R. W., and Chipman, J. W., "Earth Resource Satellites Operating in the Optical Spectrum,"in *Remote Sensing and Image Interpretation,* Fifth ed.(John Wiley and Sons, Hoboken, 2004), pp.397-490.
